# Percutaneous closure of subclavian iatrogenic injuries after central venous catheterization: a Latin American experience

**DOI:** 10.3389/fsurg.2023.1309920

**Published:** 2023-12-18

**Authors:** Carlos Eduardo Rey Chaves, Claudia Orozco, Eduardo Posada, María Gómez Zuleta, Ernesto Fajardo, Vladimir Barón, Oscar Geovanny Hernández Rodríguez

**Affiliations:** ^1^ Estudiante de Posgrado Cirugía General, Facultad de Medicina, Pontificia Universidad Javeriana, Bogotá, Colombia; ^2^ Cirugía General, Facultad de Medicina, Pontificia Universidad Javeriana, Hospital Universitario San Ignacio, Bogotá, Colombia; ^3^ Cirugía Vascular Periférica, Facultad de Medicina, Pontificia Universidad Javeriana, Hospital Universitario San Ignacio, Bogotá, Colombia

**Keywords:** subclavian injuries, percutaneous closure, outcomes, Latin America, minimally invasive

## Abstract

**Introduction:**

Arterial injuries following central venous catheterization (CVC) range between 0.1%–2.7%. The open surgical approach could be related to increased rates of morbidity and mortality. Vascular closure devices (VCD) are often used for the management of these patients with a success rate of up to 80%.

**Objectives:**

Describe our experience in managing arterial vascular injuries following central venous catheterization with Perclose ProGlide (Abbott Vascular IncSanta Clara, CA, USA).

**Methods:**

A retrospective review of all patients over 18 years old who underwent percutaneous closure of arterial injuries following central venous catheterization in our center between January 2018 and May 2023 was included and reported with a 90-day follow-up.

**Results:**

3 Patients were included, in all cases, access to the CVC were right with a subclavian artery injury. Ultrasound and fluoroscopy guide was used in all cases. For the 3 cases, a percutaneous technique using Perclose ProGlide (Abbott Vascular IncSanta Clara, CA, USA) was performed. With a 100% success rate, and no complications evidenced after 90 days of follow-up.

**Conclusion:**

Inadvertent arterial catheterization it's a non-negligible complication after CVC placement. VCD could be considered a safe and feasible approach for the management of these traumatic injuries.

## Introduction

First described by Aubaniac in 1952 central venous catheterization (CVC) it's a worldwide procedure performed by routine in a variety of clinical situations such as critically ill patients, patients who require nutritional support, and patients with oncologic conditions requiring chemotherapy (1, 2). For that purpose, jugular, subclavian, or femoral access is described with acceptable morbidity and mortality rates (1, 2). Regarding this, arterial puncture, and perforation are uncommon complications after central venous catheterization reported between 0.1%–2.7% (1, 2), nevertheless could lead to lethal consequences in up to 30% of the cases (1, 2) and penetration to supra-aortic arteries could increase the rate of complications with an increased mortality risk (1–3).

Management of these injuries includes manual compression, in some cases related to cerebrovascular events in cases of carotid compromise, or in femoral or subclavian sites could be non-successful and lead to the delay of definitive management (1–4). For that reason, surgical management, it's described and in recent years endovascular approach is gaining popularity due to comparable successful rates, with lesser morbidity and mortality compared with an open approach (1–4).

Percutaneous closure techniques are described as an alternative approach for these injuries, even “off-label”, some authors such as Lorenzo et al. (4) report acceptable successful rates with lesser morbidity and mortality rates; this data is in line with the one reported by Kania et al. (3). Therefore, our aim it's to describe the technique of percutaneous closure of arterial injuries following arterial catheterization during CVC and our experience in Colombia.

## Methods

Following Institutional Review Board approval and SCARE guidelines (5) a retrospective review of all patients over 18 years old who underwent percutaneous closure of arterial injuries following central venous catheterization in our center between January 2018 and June 2023, was registered. All patients underwent computed tomography scan and/or echography to confirm the localization of the catheter and possible associated complications. Arteriography was performed in the preoperative and postoperative periods. Follow-up was evaluated at 90 days with sonography evaluation. Informed consent was filled in prior to publication. Ethical compliance with the Helsinki Declaration, current legislation on research Res. 008430-1993 and Res. 2378-2008 (Colombia), and the International Committee of Medical Journal Editors (ICMJE) were ensured under our Ethics and Research Institutional Committee (IRB) approval.

## Technique description

**Figure 1:**
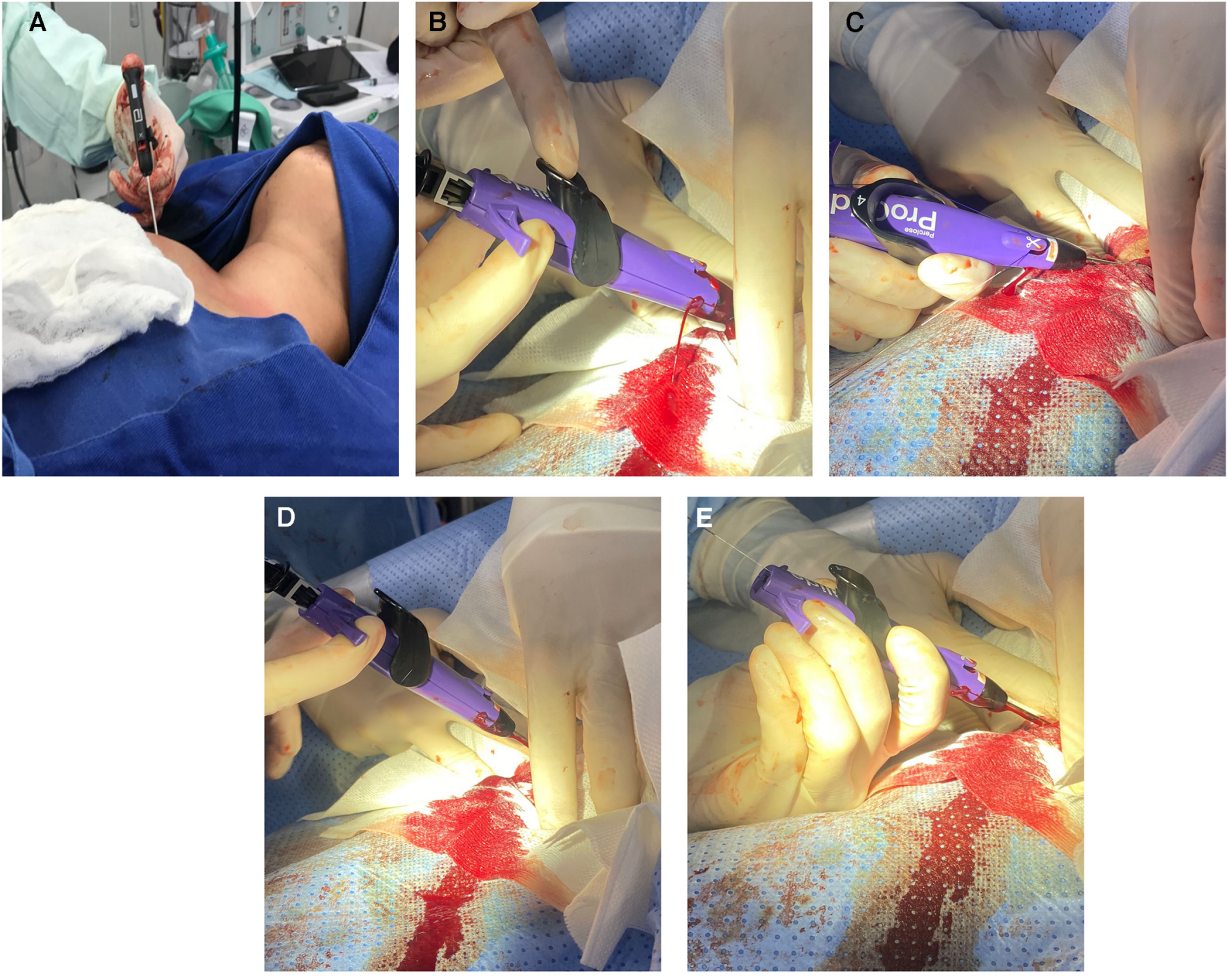
(**A**) PercloseProglide advance (**B**) recognition of injury (bleeding) (**C**) blue suture pulled out (**D**) knot positioning (**E**) white suture pulled out, knot finalized.

CVC insertion technique in our academic institution varies between surgeon experience between external venous dissection, echography guided, and using anatomic landmarks. When the surgical team confirmed the arterial puncture, arteriography and percutaneous closure were performed in the angiography surgical room. A hydrophilic guidewire was advanced by Seldinger technique into the catheter, and when correctly placed with a fluoroscopy guide, the catheter was removed. A Perclose Proglide device was advanced over the wire and deployed using the device alert and fluoroscopy guide. When positioned, the blue rail suture was pulled out, and then the knot was advanced to the injured artery until the bleeding was controlled. At that moment, the white rail suture was pulled out and the final knot was placed and secured (Figure 1). After the procedure was finalized, arteriography and/or echography were performed with no evidence of residual and/or intrathoracic bleeding.

## Results

**Figure 2:**
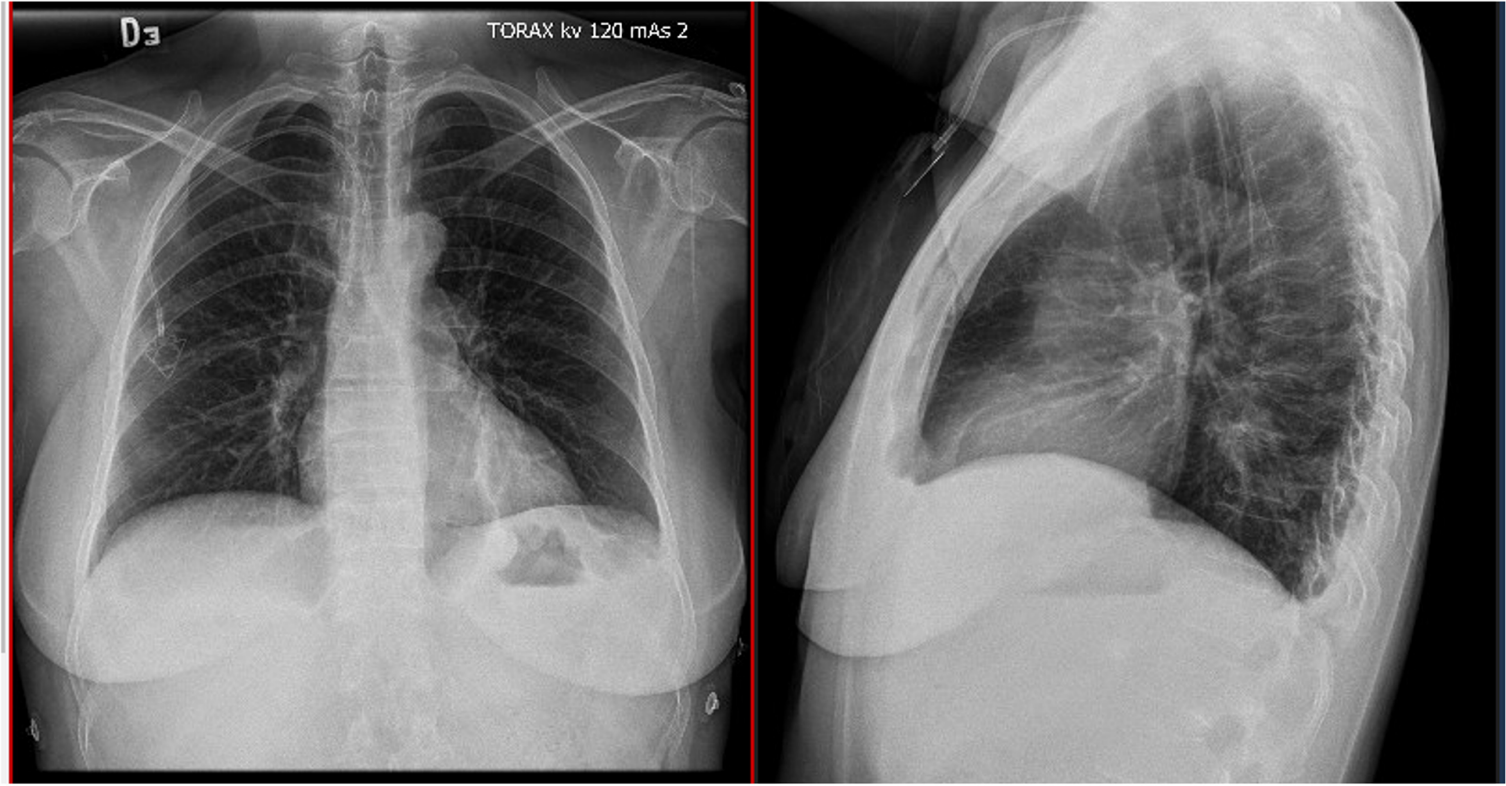
Thoracic x-ray with catheter with an arterial route.

**Figure 3:**
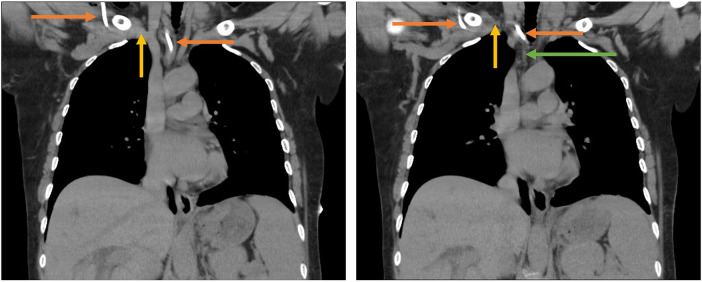
Tomography scan with evidence of the intra-arterial position of the catheter.

A total of 3 patients were included, 2 females and 1 male. In all cases, CVC was positioned under non-emergent conditions with oncologic diagnosis. Just one patient had a previous history of CVC placement. In all cases CVC was placed using the anatomic landmarks technique. Diagnosis of arterial puncture was performed by identification of arterial flow before introducing the dilator, or by postoperative thoracic x-ray, and confirmed using computed tomography scan and/or echography (Figures 2, 3). In all cases when suspected and/or confirmed arterial injury, vascular surgery group evaluation was requested immediately. In two cases there is only guidewire advance to the artery, and in one case dilator was introduced as well. Arteriography was performed for all cases, with evidence of a catheter placed in the subclavian artery in 2 cases, and in one case the catheter was advanced to the brachiocephalic trunk. There is no evidence of intrathoracic bleeding or associated thrombosis. In just one case, the patient presented with associated pneumothorax <10%. Due to intraoperative findings and clinical stability, percutaneous closure approach was preferred using ProGlide Perclosed 8 Fr with 100% success rate, and arteriography was performed at the end of the procedure with no evidence of intrathoracic bleeding, pleural effusion, or other complications. For all cases in the hospital length of stay was 1 day and there is no evidence of morbidity at 30–60 and 90 days with echography performed at 90 days with no evidence of pseudoaneurysms, arterial or venous thrombosis, or arteriovenous fistula (See Table 1).

**Table 1:** Cases summary.

	Case 1	Case 2	Case 3
Gender/age	Female, 49 years old.	Male, 51 years old.	Female, 51 years old.
Preoperative diagnosis	Ovarian cancer with peritoneal progression	Stage IV left colon cancer	Stage IV Gastric cancer
Previous central venous catheter	Yes	No	No
Arterial injury identification	Guide advance → Arterial flow	Guide advance → Arterial flow	Guide and dilator advance
Hemodynamic compromise	No	No	No
Diagnosis method	Tomography/Arteriography	Ecography/Arteriography	Tomography/Arteriography
Localization	Subclavian artery	Subclavian artery—Second portion	Subclavian artery—Second portion (Catheter advanced at the brachiocephalic trunk
Associated complications	No	No	Pneumothorax < 10%
Percutaneous closure	ProGlide Perclosed 8 Fr	ProGlide Perclosed 8 Fr	ProGlide Perclosed 8 Fr
Follow up	90 days—No complications	90 days—No complications	90 days—No complications

## Discussion

Through the years, the placement of CVC is increased, reaching more than 5 million per year in the United States (6), with a range of possible complications between 2%–20% (6). Morbidity includes pneumothorax, hemothorax, arterial puncture, or vascular injuries that could reach 9% in some studies (1–6). Even with the use of image-guide techniques, complications could appear in up to 3% of the cases (7). Depending on the localization, subclavian, carotid, brachiocephalic, femoral, or aortic branch the complications could be a hematoma, pseudoaneurysm, arterial dissection, stroke, and in rare cases (0.2%–2.5%) death (1–7).

The management of these injuries should be prompt independent of the vessel size, due to the inherent risk of pseudoaneurysm, arterio-venous fistula development or death (4–7).

Initial manual compression is described for these patients, although this method could be ineffective regarding the anatomical considerations of subclavian vessels (4–7), and in patients in which the injured vessel is the carotid, stroke is a non-negligible risk (4–8). As well other possible complications described following successful manual compression for the management of this condition, are pseudoaneurysms and arteriovenous fistula. These complications are more frequent when compared with percutaneous closure according to the present literature (4, 9).

Surgical management is widely described, open approaches using large thoracotomy or even sternotomy depending on the localization of the injury could be related to increased morbidity and mortality risk, considering that these patients in most cases have critical or oncologic conditions that increase the morbidity and mortality (3). For that reason, endovascular techniques have been described with an acceptable success rate with a lesser risk of complications (3). Since the first description in the 90's, the use of vascular closure devices (VCD) is increasing in an exponential way, due to the quick resolution of the injury (about 5 min in comparison with 20–30 min of manual compression). VCD is divided into active or passive devices, according to the material employed to achieve hemostasis. The Passive VCD often uses prothrombotic materials vs. active VCD that use sutures, clips, or collagen plug devices and is related to an increased success rate in some case series (1, 4, 6, 8).

The Perclose ProGlide (Abbott Vascular IncSanta Clara, CA, USA) it's a suture mediated active VCD that closes the vessel injury using single monofilament polypropylene, and it's designed for the closure of femoral puncture after interventional procedures (10). Although the initial indications of the ProGlide device do not include traumatic vascular injuries or another vessel than the common femoral artery; Kania et al. support the favorable outcomes of the percutaneous closure of supra-aortic vascular injuries with a success rate of up to 80% for all devices, and up to 85% for Perclose ProGlide independent of the sheath size (3). One of the advantages of this device is the possibility of more than one deployment of the suture even if the initial bleeding isn't controlled properly (3). When bleeding control isn't achieved, other endovascular techniques should be considered such as transfemoral balloon occlusion, or the placement of a stent graft to achieve prompt hemostasis (3).

Nevertheless, even with successful management after percutaneous closure of arterial injuries, sequelae such as arteriovenous fistula or pseudoaneurysms have been described (9) and appear due to the large injury of the arterial wall, however, these complications are infrequent and related in most of the cases with conservative treatment, and for that reason large follow-up is recommended in order to identify these complications (9), and when presented endovascular treatment should be performed (9). Our population has a 100% success rate using this device with no complications after 90 days of follow-up. All patients underwent arteriography and echography evaluation to dismiss further complications in our population.

Our paper increases the evidence regarding the use of percutaneous closure techniques for vascular traumatic injuries following CVC placement, with a low complication rate and a high rate of success and it's the first outcome report in Colombia.

## Conclusion

Vascular closure devices could be considered a safe and feasible approach for traumatic vascular injuries following CVC placement, with a minimally invasive approach reducing the morbidity and mortality of open approaches. Further prospective studies with a larger sample size should be performed to evaluate the safety and effectiveness of this approach.

## Data Availability

The raw data supporting the conclusions of this article will be made available by the authors, without undue reservation.
